# Supplementary material for: Impacts of hemispheric solar geoengineering on tropical cyclone frequency
Source: Nat Commun. 2017 Nov 14;8:1382. doi: 10.1038/s41467-017-01606-0 (PMC5686195; doi:10.1038/s41467-017-01606-0)
Supplement: Supplementary file 1 — Supplementary Information [file 41467_2017_1606_MOESM1_ESM.pdf]

Supplementary Note 1. How do atmospheric aerosols affect North Atlantic tropical cyclone (TC) frequency?

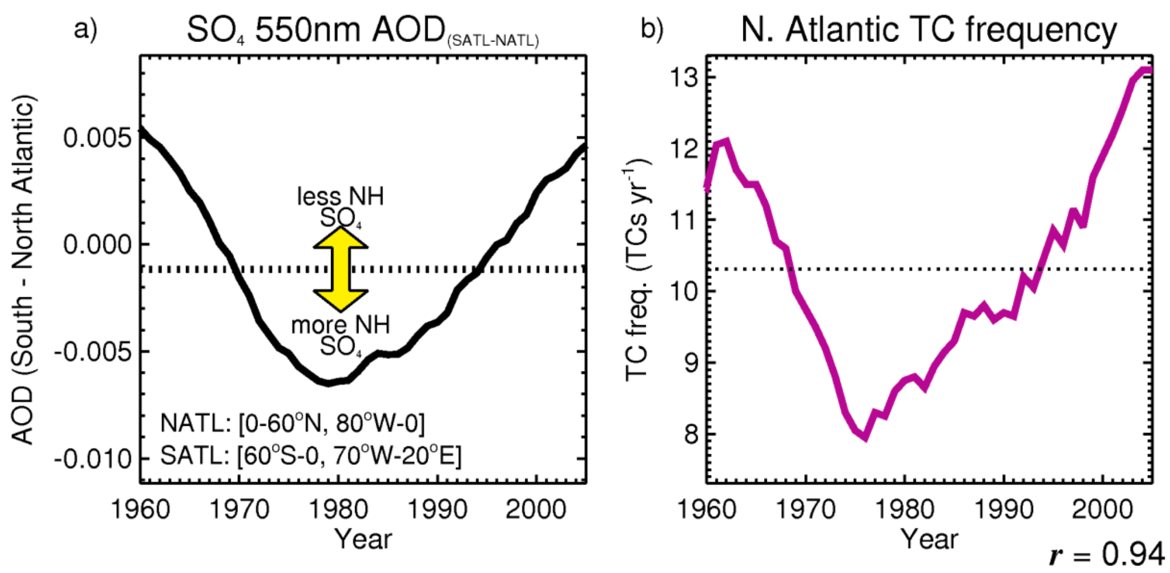

Supplementary Figure 1. Exploring the relationship between asymmetrical aerosol burdens and North Atlantic Tropical Cyclone activity. a, Sulfate (SO_4) 550nm aerosol optical depth (AOD) time-series of difference between the North Atlantic basin and South Atlantic basin in HadGEM2-ES HIST simulations, **b,** simulated North Atlantic TC frequency. Both indices are smoothed by 10 y simple moving average

Various studies have investigated the relationship between atmospheric aerosols and North Atlantic TC frequency [1,2,3,4]. Ref. 3 in particular found a strong correlation between active (inactive) TC periods and periods of abated (enhanced) anthropogenic aerosol emissions over the 20th century. Supplementary Figure 1 shows the difference between sulfate (SO_4) 550nm aerosol optical depth (AOD) in the South Atlantic ocean basin [0°-60°S, 70°W-20°E] and the North Atlantic ocean basin [0°-60°N, 80°W-0°] for the period 1960-2005 (Supplementary Fig. 1a), and the corresponding North Atlantic annual TC frequency as determined using TRACK (Supplementary Fig. 1b). Both sets of data are derived from the HadGEM2-ES HIST simulations and have been smoothed by a 10 y simple moving average. The correlation between the variables is striking at 0.95, suggesting a strong positive linear relationship. This result suggests that periods of high aerosol concentrations over the North Atlantic ocean (associated with elevated anthropogenic emissions) acted to partially attenuate North Atlantic TC frequency in the recent historical period. Note that this result simply reiterates the findings of Ref. 3, suggesting that the cooling impact of aerosols over the Atlantic, whether

tropospheric or stratospheric, have implications for Atlantic storm frequency. However, we acknowledge that correlation does not prove causality.

Supplementary Note 2. Motivation for using different TC identification methods

A key deficiency of explicitly simulated TCs from GCMs is the inability of coarse-resolution GCMs to resolve observed storm intensities and synoptic features related to cyclogenesis [5]. However, explicitly simulated TCs are able to alter the ambient meteorology which may then modify the storm's features; a feedback which is not incorporated by statistical or dynamical downscaling methods [6]. TC proxies are useful tools for relating storm activity to meteorological conditions and are readily applicable to observations and reanalysis/climate model output, but do not account for changes to TC tracks or intensity [3,6]. The statistical-dynamical downscaling model developed by Ref. 7 uses meteorological data (daily horizontal wind fields and thermodynamic variables on pressure levels) to drive a beta-and-advection (BAM) model, with tracks then integrated using an intensity model that is also coupled to meteorology. The merits of this model include its high resolution (using angular momentum coordinates) which capably resolves observed storm intensities and frequency [7]. However, the model is not coupled to the meteorology, which neglects any feedback that may occur between the TCs and climate.

Supplementary Note 3. Are the TC frequency changes statistically significant?

			Raw TC frequency: *p*-values<br>Control: 1950-2000 (HIST)	
Scenario	Period	Av. TCs yr^{-1}	WRST	t-test
RCP4.5	2020-2070	9.7	**0.17**	**0.26**
	2050-2070	9.2	**0.07**	**0.13**
	2060-2070	8.6	0.018	**0.057**
	2060-2089	8.7	1.5×10^{-3}	2.1×10^{-3}
G4	2020-2070	11.2	**0.12**	**0.15**
	2050-2070	11.2	**0.22**	**0.39**
	2060-2070	11.7	**0.07**	**0.2**
G4NH	2020-2070	7.6	3.6×10^{-6}	3.1×10^{-6}
	2050-2070	7.8	2.3×10^{-4}	1.8×10^{-4}
	2060-2070	7.8	1.8×10^{-3}	3.7×10^{-3}
G4SH	2020-2070	14.3	1.2×10^{-7}	1.1×10^{-8}
	2050-2070	13.9	9.4×10^{-4}	9×10^{-5}
	2060-2070	13.5	0.042	5.6×10^{-3}

Supplementary Table 1. Testing the significance of the changes in simulated tropical cyclones. p-values calculated from a 2-sided Wilcoxon rank sum test (WRST) and a Student’s t-test on annual TC frequency for TRACK configuration (4.5, 3.5, 4). Bold font indicates the values that are not significant at the 5% level (i.e. > 0.05).

To determine whether TC frequency changes are statistically significant with respect to HIST, we employ a Wilcoxon rank sum test (WRST) [8], which is similar to a Student’s t-test but without the underlying assumption of normally distributed data. We apply the WRST to raw TC counts and compare the HIST period with various subsets of the RCP4.5 and SAI time periods (Supplementary Table 1). Additionally, we employ a Student’s t-test to further corroborate our results, although we note that the data is not normally distributed (being count data) and therefore the WRST is the more appropriate test. It is clear from the *p*-values in Supplementary Table 1 that the northern hemisphere solar geoengineering (G4NH) and southern hemisphere geoengineering G4SH TC changes are significant at the 5% level for all time periods. Conversely, the raw TC frequency changes in the G4 and RCP4.5 scenarios are not statistically significant at the 5% level when comparing 2020-2070 with HIST.

Supplementary Note 4. Exploring statistical relationships between TC frequency and MDR precipitation, wind shear and sea-surface temperatures

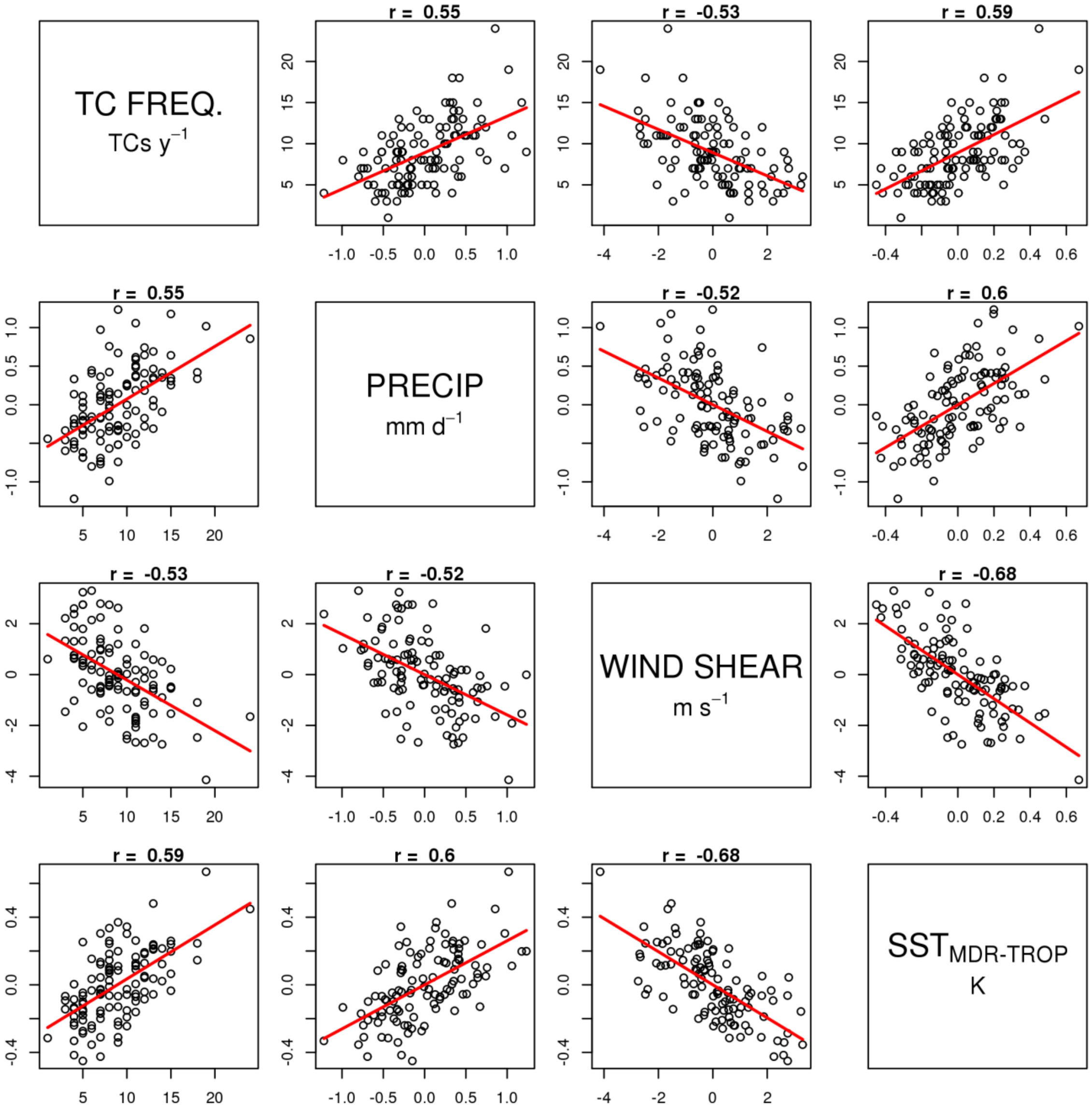

Supplementary Figure 2. Relationships between various North Atlantic climate indices and TC activity. Scatterplots of the observations and reanalysis data plotted in Fig. 7. Red lines are linear regressions (fit with lm(·) in R) and titles give Pearson correlation coefficients. Precipitation, wind shear and $SST_{MDR\text{-}TROP}$ have been normalised to zero

Supplementary Figure 2 shows scatter plots of the observations and reanalysis data in Fig. 7 with linear fits and Pearson correlation coefficients (r). It is clear that there are positive relationships between TC frequency and relative SST (r = 0.59) and between TC frequency and precipitation (r = 0.55). Additionally, there is a negative relationship between TC frequency and wind shear (r = -0.53). Consequently, due to the transitive principle, there are similarly strong relationships between precipitation, wind shear, and relative SST.

To complete this quantitative investigation of the relationships between TC frequency and the climatic indices, it is useful to develop statistical models. Specifically, we adopt the same approach as Ref. 9 and utilise a Poisson distribution framework.

$$P(N_i = k|\ \Lambda_\mathrm{i}) = \frac{e^{-\Lambda_\mathrm{i}}\Lambda_\mathrm{i}^\mathrm{k}}{k!}$$

$$\Lambda_\mathrm{i} = \exp(\beta_0 + \beta_1 x_{1,i} + \cdots)$$

The first equation describes the probability of attaining an integer value 'k' as a function of rate parameter Λ. The second equation relates the rate parameter to the covariates x, where the subscript i denotes a single realisation. The mean and variance of the Poisson distribution are both equal to Λ. In reality the variance of a random variable is often different to its mean which can lead to over or underdispersion when using a Poisson distribution. Consequently other statistical distributions such as the Negative Binomial distribution are often used.

	Precipitation	Wind Shear	$SST_{MDR\text{-}TROP}$	SST_{MDR} + SST_{TROP} [Villarini *et al.*, 2010]
β_0	2.16 (0.03)	2.16 (0.03)	2.16 (0.03)	2.16
β_1	0.48 (0.06)	-0.16 (0.02)	1.2 (0.15)	1.05
β_2	-	-	-	-1.12
Degrees of freedom of fit	2	2	2	3
Mean (res)	-0.01	0.01	-0.01	0.02
Variance (res)	1.14	1.16	1.03	1.10
Skewness (res)	0.03	0.11	0.09	0.08
Kurtosis (res)	2.54	3.35	2.26	2.44
Filliben (res)	0.997	0.994	0.994	0.994
AIC	571.2	574.1	562.2	565
SBC	576.6	579.4	567.6	575.1

Supplementary Table 2. Details of statistical relationships between various climate covariates and TC activity. *Statistical models (Poisson distributions) – covariates (β) with standard errors in brackets, properties of normalised randomised quantiles (mean, variance, skewness, Filliben coefficient), and AIC/ SBC measures of model fit*

As in Ref. 9, we build the statistical models using the 'gamlss' package in the statistical software R. We have developed 4 models: 3 single-covariate models for precipitation, wind shear, and relative SST, and 1 model with SST_{MDR} and SST_{TROP} as covariates and β values from Table 5 in Ref. 9. Supplementary Table 2 shows the values of the covariates, the

properties of the distribution of normalised randomised quantile residuals, and the Akaike Information Criterion (AIC) and Schwarz Bayesian Criterion (SBC). The AIC and SBC are measures of model fit, and incorporate penalties for the number of covariates (i.e. the degrees of freedom). Smaller AIC and SBC values indicate a better fit to the data. The normalised randomised quantile variables should be normally distributed with a mean of approximately 0 and variance of approximately 1 for an acceptable model fit. The β_1 values in Supplementary Table 2 indicate a positive relationship between MDR precipitation / $SST_{MDR\text{-}TROP}$ and TC frequency, and a negative relationship between wind shear and TC frequency. The residual distributions all appear to be close to N(0,1) with skewness ~0 and kurtosis ~ 3 as would be expected for a good model fit. Comparing the AIC and SBC values between the models indicates that $SST_{MDR\text{-}TROP}$ model provides the best fit to the data. The β_1 and β_2 values in the

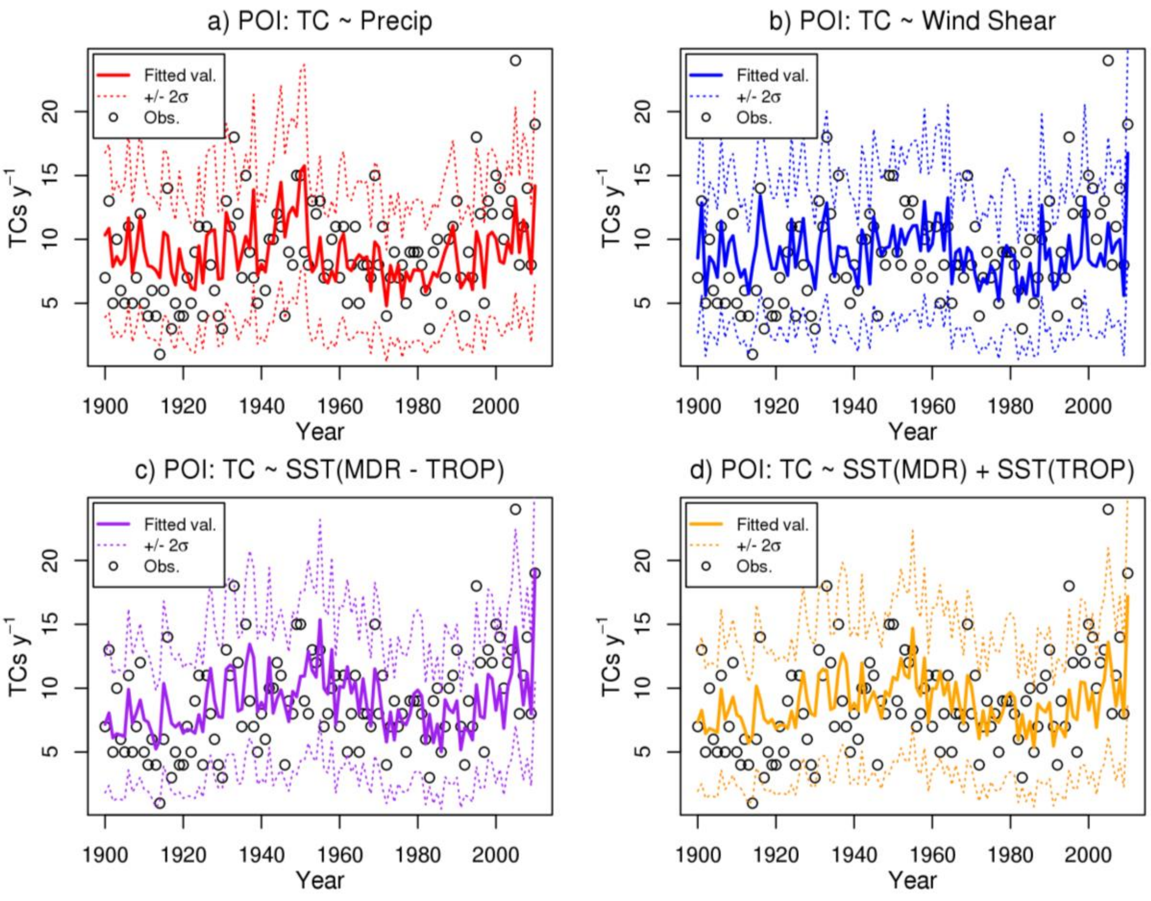

Supplementary Figure 3. Fitted TC frequency values from the statistical models. *Fitted values and +/- 2 standard deviations from the statistical models (Poisson (POI) distributions), plotted with HURDAT TC frequencies (circles) for:* ***a,*** *precipitation,* ***b,*** *vertical wind shear,* ***c,*** *hurricane main development region (MDR) and tropical-mean sea surface temperatures (SSTs) as separate covariates and* ***d,*** *relative SSTs*

SST_{MDR}+SST_{TROP} model are both significant and approximately equal to +/- 1 which gives us confidence in the use of $SST_{MDR-TROP}$ as a single covariate.

Supplementary Figure 3 shows the fitted values (i.e. Λ_i) from the statistical models plotted alongside the HURDAT TC frequencies. All of the models seem to capture the oscillating trend with peaks (troughs) in 1950 and 2010 (1900 and 1980). There seems to be little sign of overdispersion (i.e. the observations are generally within +/- 2σ) suggesting that the Poisson model is appropriate for this data [9].

Supplementary Note 5. Is the temperature response similar to El Niño?

There is no clear consensus on the impact of stratospheric aerosol on El Niño Southern Oscillation (ENSO) response, with modelling suggesting either no response [17], or SST patterns that project onto El Niño [18] or La Niña [19]. However, a growing body of evidence suggests that volcanic eruptions that preferentially load the northern hemisphere stratosphere with aerosol, lead to SST anomalies that resemble those of naturally occurring El Niño [20]. Here we use the frequently used Niño3.4 metric [21] to assess whether our simulations give SST patterns that project onto ENSO features.

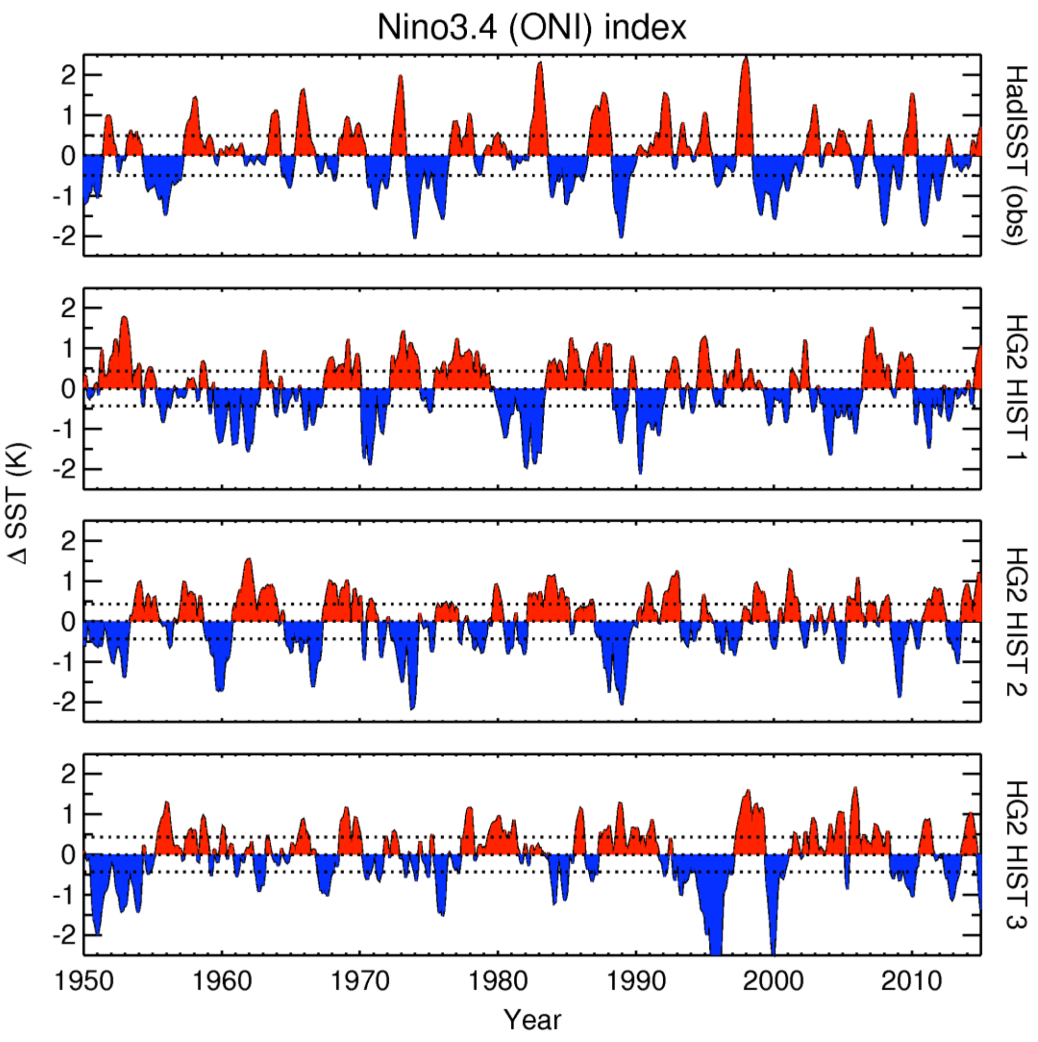

Supplementary Figure 4. The Niño3.4 index derived for HadISST observations and HadGEM2-ES historical (HIST) simulations. The dashed horizontal lines represent the threshold for defining neutral, El Niño and La Niña events. HadISST gives event frequency over the 1950-2000 period of 41% neutral, 27% El Niño and 31% La Niña using a threshold of ±0.5 K, while the ensemble mean from HIST gives 42% neutral, 28% El Niño, and 30% La Niña using a threshold of ±0.43 K.

The frequency and duration of ENSO events in the HIST simulations is similar to that derived from the HadISST data set (Supplementary Fig. 4) [22]. Niño3.4 indices are typically derived assuming El Niño/La Niña when the mean SST anomaly over the Niño3.4 region (5°S-5°N, 170°-120°W) exceeds a threshold of ±0.5 K in magnitude in 5 successive months in a calendar year. The mean of the ensemble from HadGEM2-ES simulations is slightly weaker than in the observations, but gives very similar values to the observations when the threshold is ±0.43 K, a value that we adopt in this study.

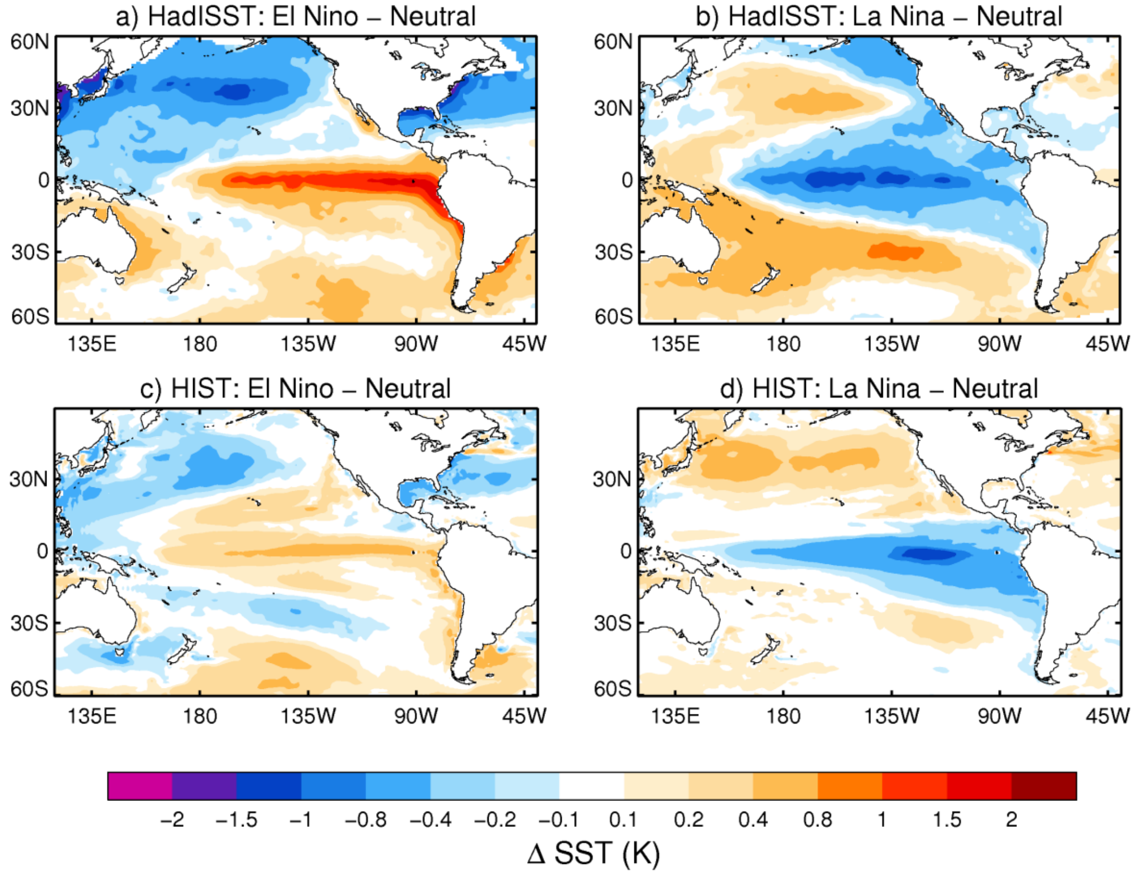

Supplementary Figure 5. Spatial distributions of El Niño and La Niña sea surface temperatures from observations and HadGEM2-ES simulations. The distribution of SST anomalies (K) over the Pacific ocean for **a,** observed El Niño, **b,** observed La Niña, **c,** modelled El Niño, **d,** modelled La Niña.

Having shown that the frequency and duration of neutral/ El Niño/ La Niña events in the HadGEM2-ES model HIST simulations are reasonable, we show the spatial distribution of the SST temperature anomalies. To emphasise the spatial distribution we chose to show normalised anomalies, where the mean SST in the region shown is zero (Supplementary Fig. 5). The distribution of the SST anomalies is similar in the HIST simulations to the observations although the strong gradient of SSTs at the equator are not evident in the model

for El Niño conditions and the cooling off the west coast of North America is not evident in La Niña conditions. However, the results above show that HadGEM2-ES does exhibit a credible ENSO performance in terms of the frequency, duration and spatial distribution particularly when compared to the biases in other global coupled atmosphere ocean models [23,24]. We therefore examine the response in these variables in our geoengineering simulations.

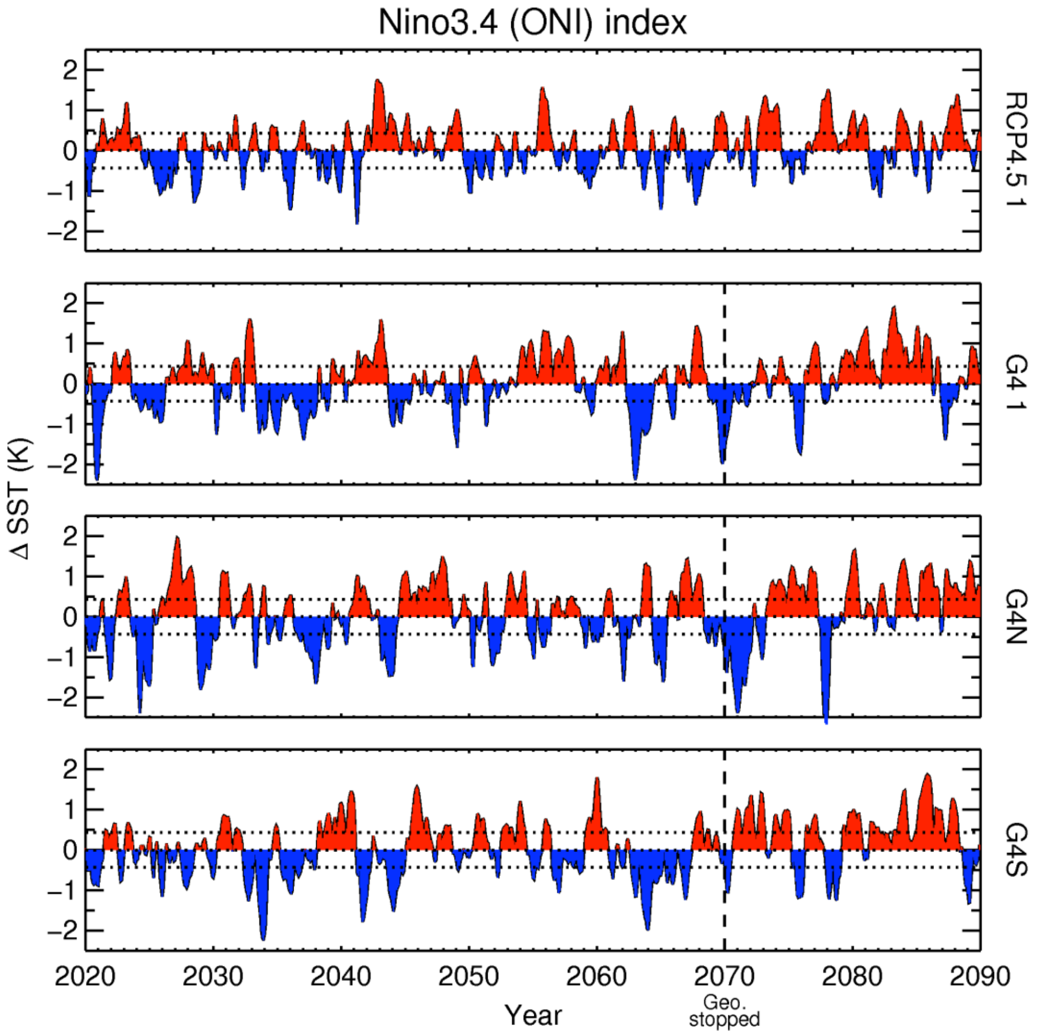

Supplementary Figure 6. The Niño3.4 index derived for RCP4.5, and the solar geoengineering simulations G4, G4NH and G4SH. The dashed horizontal lines represent the threshold for defining neutral, El Niño and La Niña events. The proportion of neutral/ El Nino/ La Nina years for the period 2020-2070 is 54/ 25/ 20 % for RCP4.5, 46/ 26/ 28 % for G4, 39/ 33/ 27 % for G4NH, and 45/ 25/ 29 % for G4SH

Supplementary Figure 6 shows that there is little change in the frequency of neutral/El Niño/La Niña events. Interestingly, the period that shows the most dominant El Niño signal in the G4 simulations is the period after 2070 when the applied geoengineering ceases. Thus,

in the HadGEM2-ES model at least, there is no evidence that stratospheric geoengineering enhances either El Niño or La Niña frequency. We note that the Niño3.4 index is very much concentrated on the equatorial regions of the Pacific (5°S-5°N), so it is instructive to look for similarities in ENSO response outside of this narrow latitudinal band.

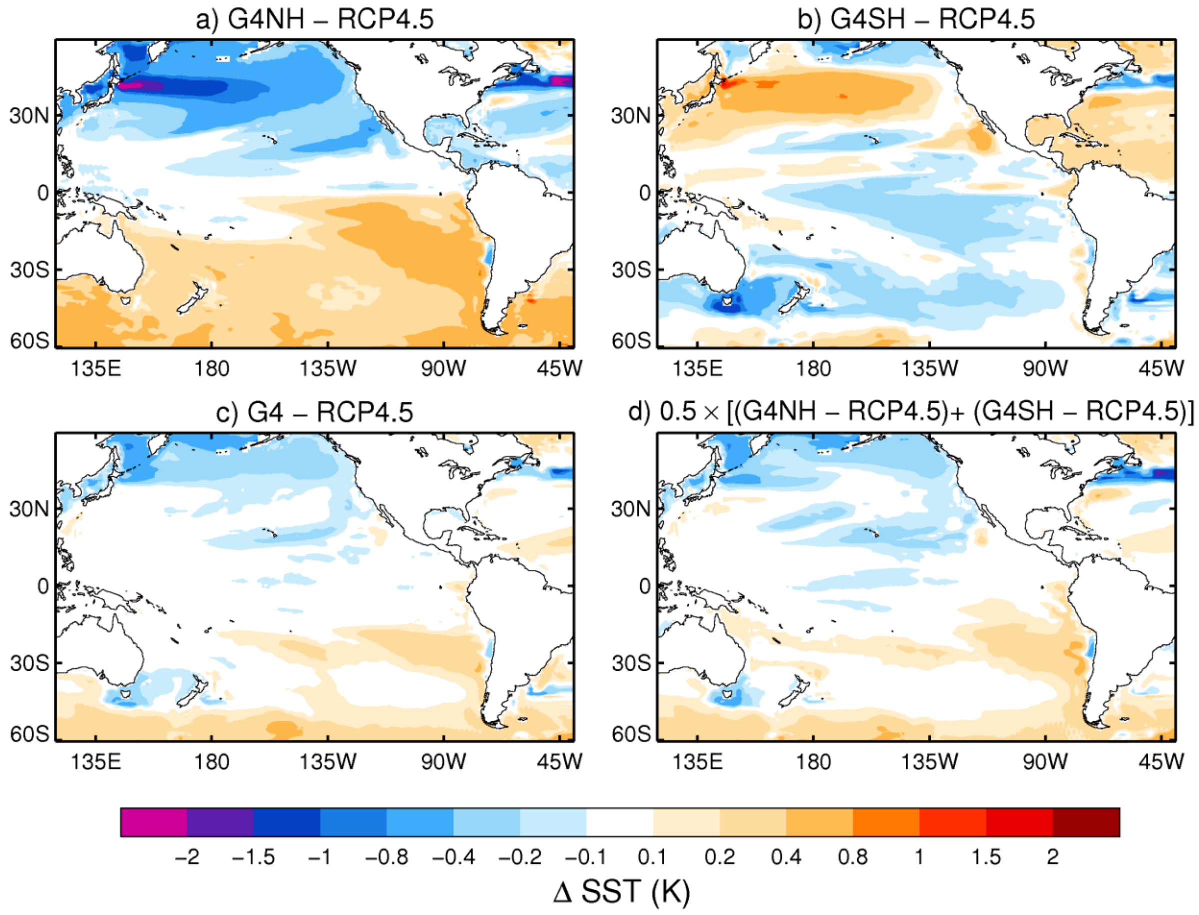

Supplementary Figure 7. Spatial distributions of sea surface temperature anomalies in the solar geoengineering simulations relative to RCP4.5. The distribution of SST anomalies (K) over the Pacific ocean induced by **a,** G4NH, **b,** G4SH, **c,** G4, **d,** a linear combination of G4NH and G4SH

Supplementary Figure 7 shows there are some considerable similarities, but also some considerable differences when compared to the modelled spatial pattern of El Niño SST response shown in Supplementary Fig. 5c. In G4NH, there is broad and consistent cooling of the northern hemisphere in comparison to the southern hemisphere, which reflects the nature of the applied northern hemispheric forcing, while the El Niño signal diagnosed in Supplementary Fig. 5c shows a much broader tropical warming with areas of extra-tropical cooling. When comparing G4SH simulations of SST to the La Niña SST response in Supplementary Fig. 5d, there are again some considerable similarities but some considerable

differences. For example the broad equatorial cold tongue is evident, but the extratropical response under G4SH differs considerably with cooling in the southern hemisphere between 30°-60°S which is not evident in the La Niña response. The G4 simulations show what is in effect a linear combination of the response in G4NH and G4SH as shown by comparing Supplementary Fig. 7c with Supplementary Fig. 7d. This indicates that a globally distributed stratospheric geoengineering scheme would have scant impact as the SST response is balanced between the northern and southern hemispheres.

Our conclusions from this analysis are that geoengineering the northern/southern hemisphere in HadGEM2-ES simulations has little impact on the commonly used Niño3.4 El Niño/La Niña frequency. While SST anomalies under G4NH/G4SH bear some similarity to El Niño/La Niña patterns particularly in the tropics, they are far from identical, particularly in the extra-tropics. Therefore one cannot assert that geoengineering under any of these scenarios would enhance or decrease El Niño/La Niña events, at least from the limited set of results from the HadGEM2-ES model.

Supplementary Note 6. Investigating changes to TC intensity using the model generated storms

TCs produced by HadGEM2-ES in the North Atlantic basin are generally less intense than those from observations or reanalyses [5,10]. This inability to reproduce the observed TC intensity distribution is a common feature of the current generation of climate models and stems from the coarse model resolutions. Indeed, higher resolution climate models are able to reproduce observed storm intensities [e.g. 'T159' resolution in Ref. 11]. Whilst this model-limitation reduces our ability to make robust conclusions regarding the impact of geoengineering on TC intensity, it is still possible to investigate changes to the overall distribution of storm intensity.

We investigate changes to the maximum sustained windspeed (MSWS) at 10m altitude. We determine the MSWS for ERA-Interim (ERA-I) [12] and the HadGEM2-ES simulations by finding the maximum windspeed within a 6° geodesic radius centred on the relative vorticity maxima at each timestep (as in Ref. 11). It is important to note that we use 6-hourly mean data for both ERA-I and HadGEM2-ES whereas the HURDAT MSWS values are provided as 1-minute maximums[1] and therefore the MSWS distributions will necessarily differ between HURDAT and ERA-I. Additionally, the HURDAT MSWS values are given to the nearest 10 knots (~5.14 m s^{-1}), whereas the ERA-I and HadGEM2-ES values are continuous. Interestingly, ERA-I and other reanalyses are presently unable to reproduce observed TC intensities due to their own model-resolution issues [13,14], which suggests that even if we were to calculate HURDAT MSWSs as 6-hourly means, ERA-I would still not reproduce the observed TC intensity distribution.

Supplementary Figure 8 shows the distribution of lifetime maximum windspeeds for each TC identified by TRACK using the criterion (6,5.5,4) for ERA-I, and (6,5.5,4) / (4.5,3.5,4) for the HadGEM2-ES historical simulations. For ERA-I and HadGEM2-ES we use a bin size of 0.2 m s^{-1}, while for HURDAT we use a bin-size of ~5 m s^{-1}. As noted above, the calculation of MSWS is different for ERA-I and HURDAT and therefore we would not expect ERA-I to reproduce the HURDAT intensity distribution in Supplementary Fig. 8. What is clear from Supplementary Fig. 8a,b is that HadGEM2-ES does not produce enough intense storms compared to ERA-I (MSWS > 15 m s^{-1}), which is reflected in the differences between the upper quantiles of the distributions. To compensate for this, we have plotted the normalised

[1] http://www.aoml.noaa.gov/hrd/hurdat/newhurdat-format.pdf

windspeeds (i.e. subtract the mean and divide by the standard deviation). For interest, the mean (standard deviation) MSWS in units of m s^{-1} are: 39.7 (15.1) for HURDAT, 19.2 (7.5) for ERA-I, 13.5 (2.7) for HadGEM2-ES (4.5,3.5,4) and 14.5 (2.9) at HadGEM2-ES (6,5.5,4). Supplementary Fig. 8c,d show the normalised MSWS distributions, which are much closer to HURDAT than the raw distributions but still exhibit significant differences. For instance, the HadGEM2-ES simulations exhibit smaller interquartile ranges than ERA-I, suggesting that the simulated TC intensity distribution is too concentrated around a mean value.

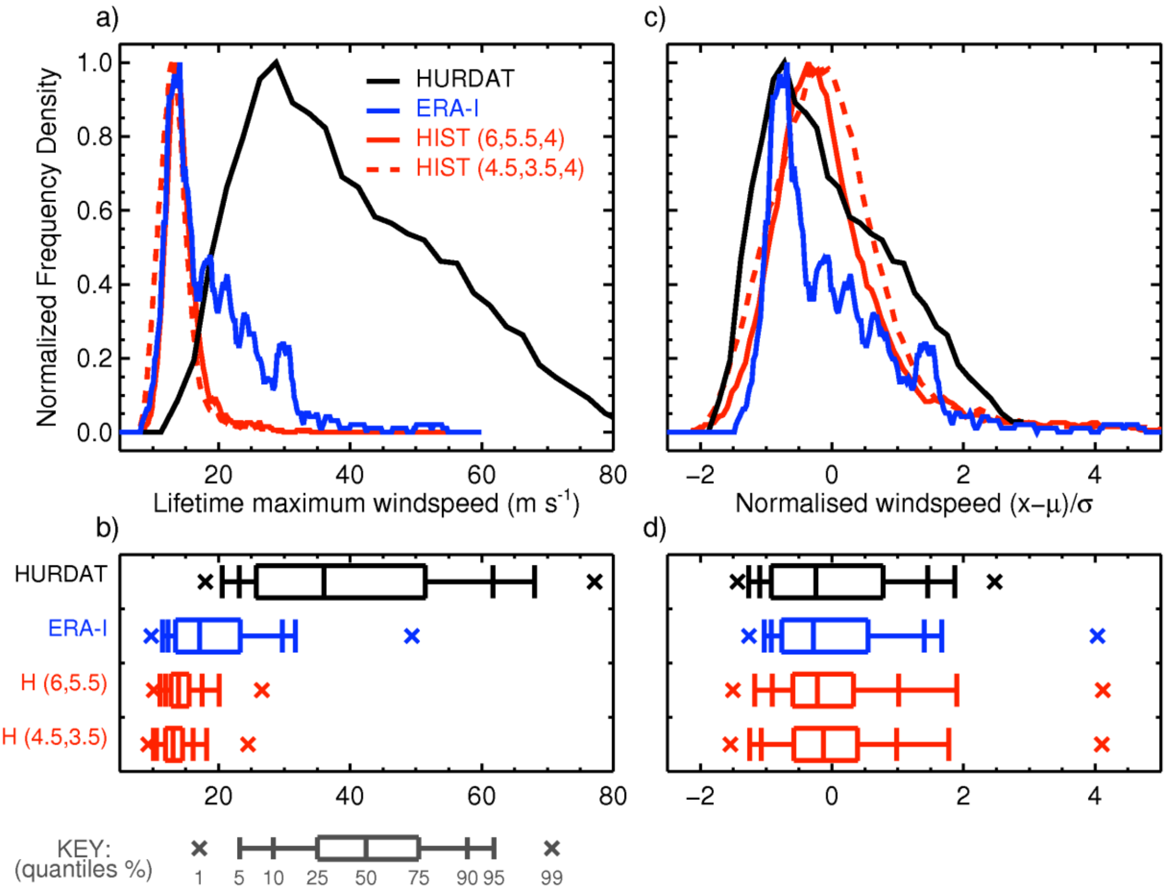

Supplementary Figure 8. Normalised frequency distributions of maximum storm intensities in the observations, reanalyses and HadGEM2-ES historical simulations. North Atlantic tropical cyclone intensity (maximum sustained windspeed) distribution, normalised so that the mode = 1, for HadGEM2-ES (H) 1950-2000 with two TRACK configurations (red), ERA-I 1979-2014 (blue), and HURDAT obs. 1950-2000 (black). **a** and **b,** Raw intensities, **c** and **d,** normalised intensities

---

Supplementary Figure 9 shows box and whisker plots of the normalised MSWS distributions from the HadGEM2-ES simulations. The upper quantiles (75, 90, and 95% - see key in Supplementary Fig. 8) in the RCP4.5 scenario are clearly greater than equivalent quantiles in HIST, suggesting a shift to more intense storms under global warming as found in many

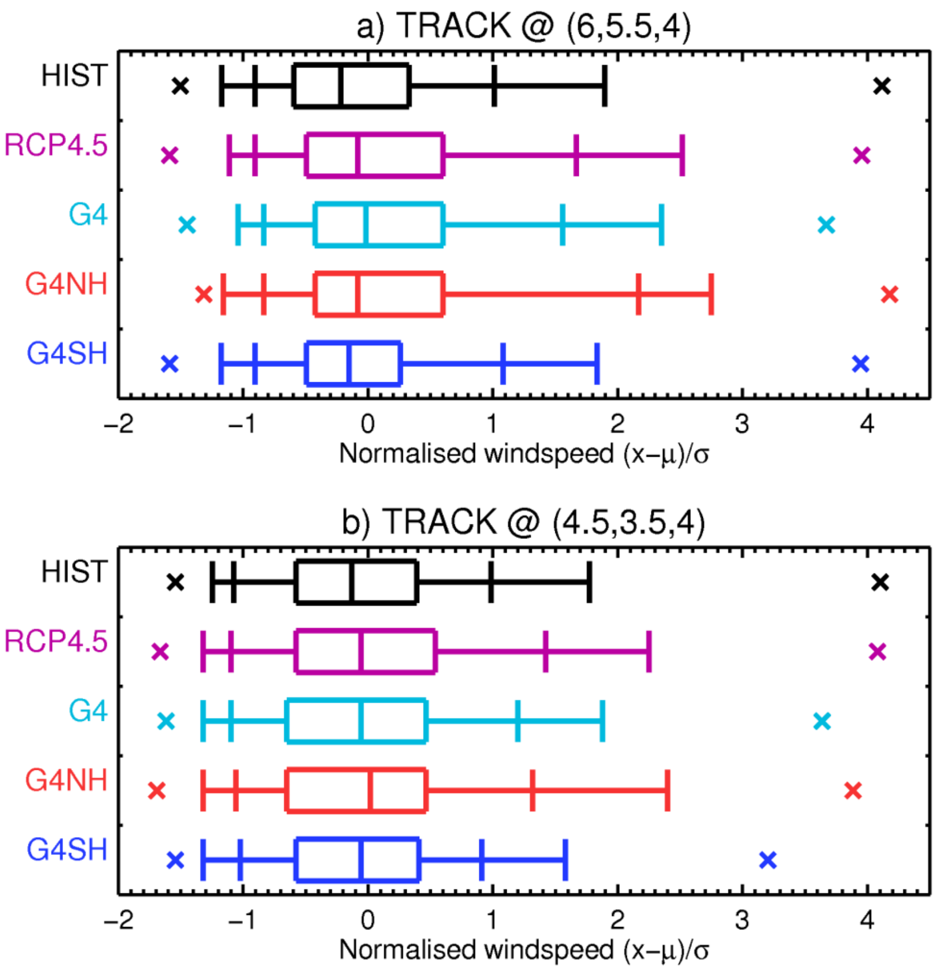

Supplementary Figure 9. Box and whisker plots of the normalised maximum-intensity distribution for two TRACK configurations. HIST is evaluated for 1950-2000 and RCP4.5, G4, G4NH, and G4SH are evaluated for 2020-2070.

---

previous studies [6,15]. The G4 and G4NH scenarios also appear to show a shift toward more intense storms, suggesting that solar geoengineering would do little to modulate increases in storm intensity despite effectively modulating global warming (e.g. Fig. 3). However, Supplementary Fig. 9 does not account for changes to TC frequency. Supplementary Figure 10 shows changes to TC frequency based on their maximum-intensity relative to the intensity distribution in the HIST era. For instance, a positive value in the first column (< $\alpha\text{-}_{\mathrm{HIST}}$ = 5 %) would indicate an increase in the number of storms that achieve a maximum-intensity that is less than the lowest 5% maximum-intensity value of the HIST distribution (i.e. indicating an increase in weak storms). It is clear that the reductions in overall storm frequency in the RCP4.5 and G4NH scenarios (Fig. 4) are mainly due to reductions in weak storms (< $\alpha\text{-}_{\mathrm{HIST}}$ = 75 %). The frequency of the most intense storms increases in all of the scenarios (> $\alpha\text{-}_{\mathrm{HIST}}$ = 95 %). G4SH exhibits increases in storm activity at all intensities, whereas G4 exhibits little

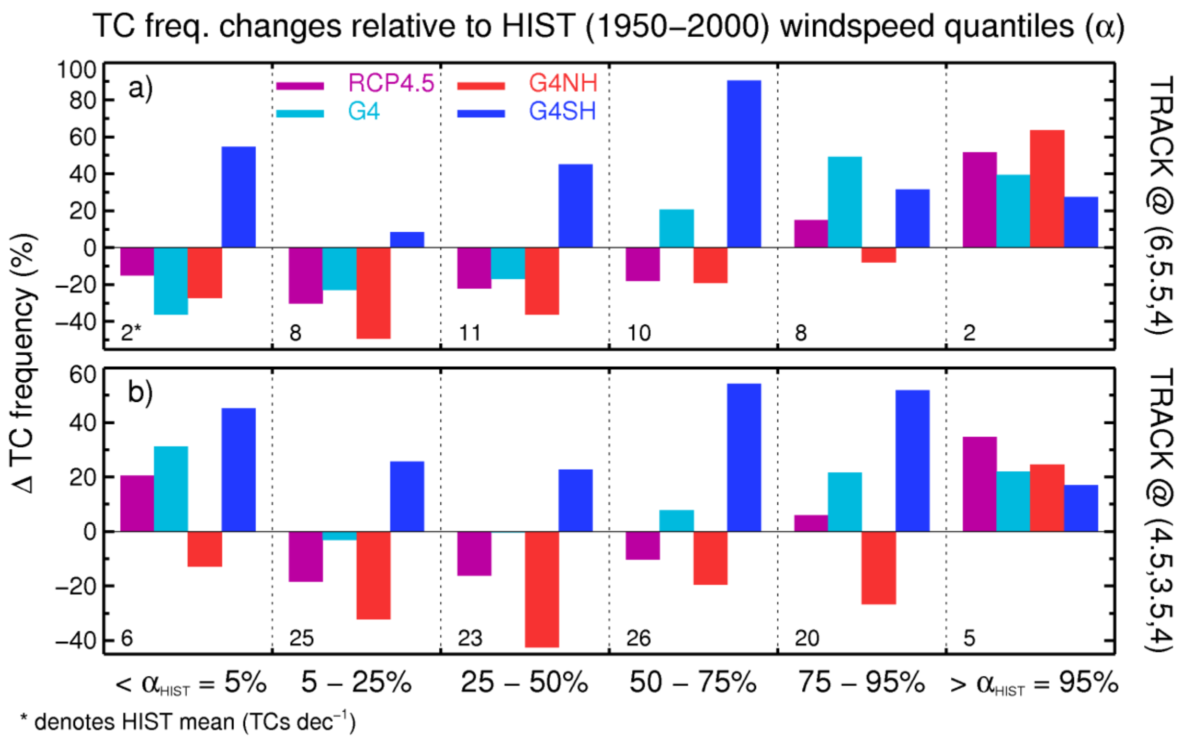

Supplementary Figure 10. TC frequency changes grouped by intensity relative to historical intensities. TC frequency changes between various percentiles of the HIST maximum-intensity distribution for two TRACK configurations: **a,** TRACK at (6,5.5,4) and **b,** TRACK at (4.5,3.5,4)

conformity between the two TRACK configurations. Note that we inject aerosol at a constant rate in the SAI simulations and the temperature rise due to enhanced greenhouse gas concentrations is not completely offset by SAI (Fig. 3a). If we had instead injected at such a rate as to stabilise global-mean temperature the changes to storm frequency and intensity in the SAI simulations might have been different.

A tendency for an overall reduction in North Atlantic TC frequency alongside an increase in the incidence of the most intense storms under global warming (RCP4.5) is a common result from previous research [6,15,16] and has also been identified here (Supplementary Figs 9 and 10). However, the inability of HadGEM2-ES to produce storms with observed intensities in the North Atlantic basin (Supplementary Fig. S8) reduces confidence in the model's ability to predict changes to the intensity distribution under global warming and SAI. In light of this, we have utilised a statistical-downscaling model based on the HadGEM2-ES climatologies to study changes to the intensity distribution (Fig. 9). The disparities between the results of the explicit storm tracking and the downscaling method should galvanise further research into storm intensity changes under SAI.

Supplementary Note 7. Are the TC changes statistically significant in the SAI and RCP4.5 simulations compared to HIST for the statistical-dynamical downscaling model?

We perform the same statistical analyses as in Supplementary Section 3 – to note, a Wilcoxon rank sum test and a Student's t-test – to assess whether TC frequency changes are significant in the results of the statistical-dynamical downscaling simulations. Supplementary Tables 3-5 show the p-values when comparing the 2020-2070 and 1950-2000 periods using a WRST (top line) and t-test (bottom line). Supplementary Table 3 compares TC frequencies (Fig. 9a), Supplementary Table 4 compares hurricane frequencies (Fig. 9b), and Supplementary Table 5 compares major hurricanes (Fig. 9c).

TC frequencies in the G4 and G4NH scenarios are not significantly different to HIST whereas all other differences are significant at the 5 % level. All of the SAI scenarios significantly reduce storm activity relative to RCP4.5, while G4 and G4NH exhibit insignificant major hurricane changes with respect to HIST at the 1 % level (Supplementary Table 5).

	HIST<br>1950-2000	RCP4.5<br>2020-2070	G4<br>2020-2070	G4NH<br>2020-2070	G4SH<br>2020-2070
RCP4.5<br>2020-2070	0<br>6.7×10^{-16}	-	-	-	-
G4<br>2020-2070	1.5×10^{-3}<br>9.5×10^{-4}	0<br>5.7×10^{-10}	-	-	-
G4NH<br>2020-2070	6.2×10^{-3}<br>8.5×10^{-3}	0<br>5.7×10^{-10}	**0.37**<br>**0.64**	-	-
G4SH<br>2020-2070	1.8×10^{-7}<br>1.8×10^{-8}	2×10^{-4}<br>2.3×10^{-4}	1.6×10^{-3}<br>1.4×10^{-3}	2.4×10^{-3}<br>1.4×10^{-3}	-

Supplementary Table 3. Testing the significance of the TC frequency changes in the statistical downscaling simulations. p-values calculated from a 2-sided Wilcoxon rank sum test (WRST) and a Student's t-test on annual TC frequency from the statistical-downscaling simulations. Bold font indicates where changes are not significant at the 5 % level

	HIST 1950-2000	RCP4.5 2020-2070	G4 2020-2070	G4NH 2020-2070	G4SH 2020-2070
RCP4.5 2020-2070	0<br>1.2×10^{-14}	-	-	-	-
G4 2020-2070	4.4×10^{-3}<br>8.3×10^{-3}	0<br>1.4×10^{-10}	-	-	-
G4NH 2020-2070	7.6×10^{-3}<br>0.01	0<br>3.8×10^{-9}	**0.48**<br>**0.89**	-	-
G4SH 2020-2070	5.4×10^{-7}<br>2.8×10^{-7}	1.6×10^{-4}<br>2.1×10^{-4}	1.5×10^{-3}<br>1.4×10^{-3}	6.6×10^{-3}<br>5.4×10^{-3}	-

Supplementary Table 4. Testing the significance of the hurricane frequency changes in the statistical downscaling simulations. The same as Supplementary Table 3 but for annual hurricane frequency

	HIST 1950-2000	RCP4.5 2020-2070	G4 2020-2070	G4NH 2020-2070	G4SH 2020-2070
RCP4.5 2020-2070	0<br>3×10^{-12}	-	-	-	-
G4 2020-2070	0.03<br>0.04	6×10^{-8}<br>1.4×10^{-8}	-	-	-
G4NH 2020-2070	8.7×10^{-3}<br>0.01	7.2×10^{-7}<br>1.9×10^{-7}	**0.27**<br>**0.59**	-	5.4×10^{-3}<br>8.1×10^{-3}
G4SH 2020-2070	1.2×10^{-6}<br>6.1×10^{-7}	1.7×10^{-3}<br>1.9×10^{-3}	4.8×10^{-4}<br>1.3×10^{-3}	5.4×10^{-3}<br>8.1×10^{-3}	-

Supplementary Table 5. Testing the significance of the hurricane frequency changes in the statistical downscaling simulations. The same as Supplementary Table 3 but for annual major hurricane frequency

Supplementary Note 8. How are TCs tracked in this study?

TC tracking is conducted using the TRACK code (vn. 1.4.7)[2] developed and utilised for a variety of similar investigations [10,11,25,26,27,28,29]. Ref. 25 provides a detailed account of TRACK's core functionality. We adopt the approach of Ref. 11 (hereafter *BE07*) here. Firstly, we determine the relative vorticity (ξ) on 850, 500, and 250 hPa vertical pressure levels from the zonal (U) and meridional (V) wind using the definition: $\xi = (1/a\times\cos(\theta)) \times (dV/d\lambda - dU\cos(\theta)/d\theta)$, where a is Earth's radius, and θ and λ are the latitude and longitude in radians respectively. U and V are provided on 6 h timesteps, and we only consider the June-November (JJASON) season here, in which most of the North Atlantic TC activity occurs. For ERA-I we use the full Gaussian resolution (H512×256) datasets on 6 h time-steps for the time-period 1979-2014 and for JJASON.

The manuscript's Methods section contains detail of the feature-tracking methodology. As with *BE07*, we test different permutations of (ξ_1, ξ_V, $n = 4$) for ERA-I [12] and HadGEM2-ES against HURDAT2 [30] annual TC frequency. For ERA-I, we find that a TC selection criterion of (6, 5.5, 4) provides a good fit to HURDAT2 data. This criterion closely resembles the *BE07* criterion of (6, 6, 4). Previous studies have found that HadGEM2-ES has a low bias in terms of TC intensity and frequency in the North Atlantic basin, which is possibly due to the coarse spatial resolution of the model [5,31,32]. In this study, we also observe this bias when using the ERA-I criterion (6, 5.5, 4) to identify TCs in the HadGEM2-ES simulations (Supplementary Fig. 11). Ref. 3 and Ref. 33 found that HadGEM2-ES is able to skilfully predict North Atlantic TC frequency trends when TC identification criteria are relaxed. As we are primarily interested in how TC frequency responds to SAI in this study, we use a similarly pragmatic approach. Specifically, we have relaxed the TC criterion to (4.5, 3.5, 4) for HadGEM2-ES simulations, which produces a similar number of TCs in HIST to the HURDAT2 observations (Fig. 4 and Supplementary Tables 6 and 7). It is important to note that the characteristics (e.g. the intensity) of the model storms will differ from those of the observed storms due to the different criteria applied. Nevertheless, the model capably produces historical TC trends (Fig. 4), which gives us confidence that this approach allows us insight into future TC frequency trends under the RCP4.5 and SAI scenarios. The correlation coefficients between the modelled TC frequencies using the two different TC selection criteria (i.e. column r in Tables S6 and S7) indicate a strong positive correlation in all

[2] http://www.nerc-essc.ac.uk/~kih/TRACK/Track.html

instances. This suggests that the results of this study would in fact be resistant to our choice of TC selection criteria.

			(4.5, 3.5, 4)		(6, 5.5, 4)		
Expt.	Ens.	Year	Mean	St. Dev.	Mean	St. Dev.	*r*
HURDAT	-	1979-2005	10.2*	4.5*	-	-	-
ERA-I	-	1979-2005	22.4	6.4	10.6	4.2	0.81
HIST	1	1979-2005	10.9	3.6	4.7	2.6	0.80
	2	1979-2005	11.9	3.6	4.4	2	0.62

Supplementary Table 6. Annual TC frequency in the observations, ERA-I reanalyses and HadGEM2-ES HIST simulations for two configurations of TRACK. TC frequency in the ERA-I era (1979-2005) for HadGEM2-ES, ERA-I and HURDAT2. *Note that the specified configuration of TRACK (i.e. (6,5.5,4) or (4.5,3.5,4)) is not relevant to the HURDAT2 observations

			(4.5, 3.5, 4)		(6, 5.5, 4)		
Expt.	Ens.	Year	Mean	St. Dev.	Mean	St. Dev.	*r*
HURDAT	-	1950-2000	9*	3.2*	-	-	-
HIST	1	1950-2000	10	2.9	3.9	2.2	0.62
	2	1950-2000	10.8	3.6	4.2	2.1	0.66
RCP4.5	1	2020-2070	9.7	3.3	3.7	1.9	0.5
	2	2020-2070	9.9	3	3.7	2.1	0.68
	3	2020-2070	9.6	3.5	3.4	2.1	0.69
G4	1	2020-2070	11.3	3.2	4.3	2.3	0.66
	2	2020-2070	11.9	4	4.9	2.1	0.77
	3	2020-2070	10.4	3.8	3.7	2.2	0.59
G4NH	-	2020-2070	7.6	2.9	3.1	1.7	0.69
G4SH	-	2020-2070	14.3	3.6	6	2.7	0.63

Supplementary Table 7. Annual TC frequency in the HadGEM RCP4.5 and solar geoengineering simulations for two configurations of TRACK. TC frequency in the 1950-2000 (HIST) and 2020-2070 periods. *Note that the specified configuration of TRACK (i.e. (6,5.5,4) or (4.5,3.5,4)) is not relevant to the HURDAT2 observations

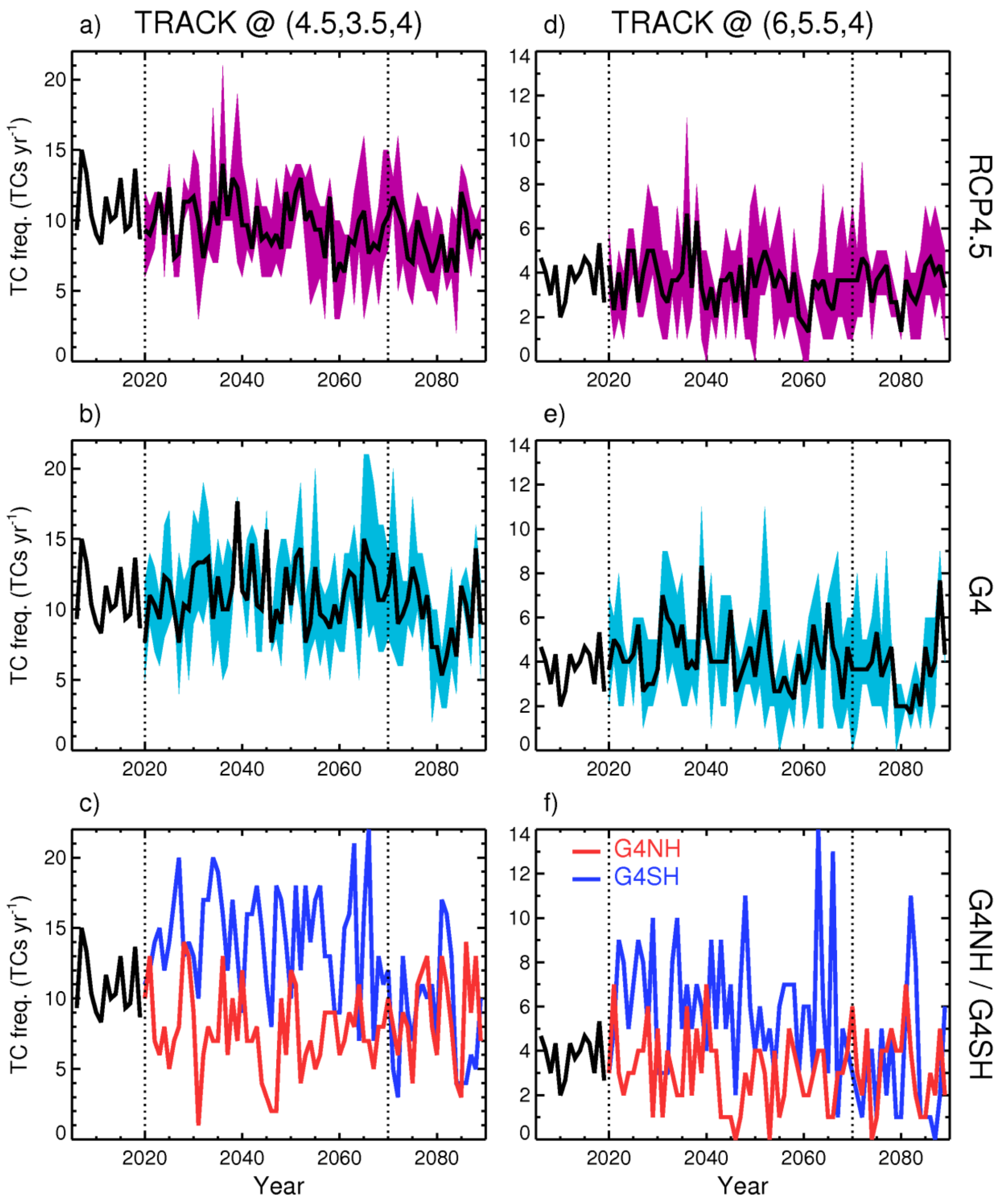

Supplementary Figure 11. Time-series of raw annual TC frequency determined using two TC selection criteria. a-c, TRACK @ (4.5,3.5,4) and **d-f,** TRACK @ (6, 5.5, 4). Purple and light-blue shading indicate the range of RCP4.5 and G4 values respectively

Supplementary Figure 11 shows the raw TC counts for the RCP4.5, G4, G4NH and G4SH simulations using the two TC criteria. It is clear that G4SH produces significantly more TCs per year than G4NH (from 2020-2070) for both TC selection criteria.

Supplementary References

[1] Emanuel, K., & Mann, M., Atlantic hurricane trends linked to climate change, *EOS*, 87, 24, 233–244., (2006)

[2] Wang, C., Dong, S., Evan, A. T., Foltz, G. R., & Lee, S.-K., Multidecadal covariability of North Atlantic sea surface temperature, African dust, Sahel rainfall, and Atlantic hurricanes, *J. Clim.*, 25, 5404–5415, (2012)

[3] Dunstone, N. J., Smith, D. M., Booth, B. B. B., Hermanson, L., & Eade, R., Anthropogenic aerosol forcing of Atlantic tropical storms, *Nature Geosci.*, 6, 534–539, (2013)

[4] Chiacchio, M., Pausata, F. S. R., Messori, G., Hannachi, A., Chin, M., Önskog, T., Ekman, A. M. L., & Barrie, L., On the links between meteorological variables, aerosols, and tropical cyclone frequency in individual ocean basins, *J. Geophys. Res. Atmos.*, 122, (2017)

[5] Camargo, S. J., Global and regional aspects of tropical cyclone activity in the CMIP5 models. *J. Clim.*, 26, 9880–9902, (2013)

[6] Emanuel, K. A., Downscaling CMIP5 climate models shows increased tropical cyclone activity over the 21st century, *Proc. Natl. Acad. Sci.*, 110(30), 12,219–12,224., (2013)

[7] Emanuel, K., Ravela, S., Vivant, E., & Risi, C., A statistical deterministic approach to hurricane risk assessment, *Bull. Am. Meteorol. Soc.*, 87, 299–314, (2006)

[8] Wilcoxon, F., Individual Comparisons by Ranking Methods, *Biometr. Bull.*, 1, 6, 80-83., (1945)

[9] Villarini, G., Vecchi, G. A., & Smith, J. A., Modelling of the dependence of tropical storm counts in the North Atlantic basin on climate indices, *Mon. Weather Rev.*, 138, 2681–2705, (2010)

[10] Strachan, J., Vidale, P. L., Hodges, K., Roberts, M., & Demory, M.-E., Investigating global tropical cyclone activity with a hierarchy of AGCMs: The role of model resolution, *J. Clim.*, 26, 133–152, (2013)

[11] Bengtsson, L., Hodges, K. I., & Esch, M., Tropical cyclones in a T159 resolution global climate model: comparison with observations and re-analyses, *Tellus A*, 59 (4), 396-416., (2007)

[12] Dee, D. P, *et al.*, The ERA-Interim reanalysis: configuration and performance of the data assimilation system. *Q.J.R. Meteorol. Soc.*, 137: 553–597., (2011)

[13] Murakami, H., Tropical cyclones in reanalysis data sets, *Geophys. Res. Lett.*, 41, 133–2141, (2014)

[14] Camp, J., Roberts, M., MacLachlan, C., Wallace, E., Hermanson, L., Brookshaw, A., Arribas, A. and Scaife, A. A., Seasonal forecasting of tropical storms using the Met Office GloSea5 seasonal forecast system, *Q. J. R. Meteorol. Soc.*, 141, 2206–2219, (2015)

[15] Knutson, T. R., *et al.*, Global projections of intense tropical cyclone activity for the late 21st century from dynamical downscaling of CMIP5/RCP4.5 scenarios, *J. Clim.*, 28(18), 7203–7224., (2015)

[16] Walsh, K. J. E., *et al.*, Hurricanes and climate: The U.S. CLIVAR working group on hurricanes, *Bull. Am. Meteorol. Soc.*, 96(6), 997–1017, (2015)

[17] Robock, A., Volcanic eruptions and climate, *Rev. Geophys.*, *38*(2), 191–219., (2000)

[18] Maher, N., McGregor, S., England, M. H., and Gupta, A. S., Effects of volcanism on tropical variability, *Geophys. Res. Lett.*, *42*(14), 6024–6033, (2015)

[19] McGregor, S., and A. Timmermann, The Effect of Explosive Tropical Volcanism on ENSO, *J. Clim.*, *24*(8), 2178–2191, (2011)

[20] Pausata, F.S., Chafik, L., Caballero, R., & Battisti, D. S., Impacts of high-latitude volcanic eruptions on ENSO and AMOC. *Phil. Trans. R. Soc. A*, *112*(45), pp.13784-13788., (2015)

[21] Trenberth, K. E., The definition of El Nino., *Bull. Am. Meteorol. Soc* 78, 12. 2771-2777., (1997)

[22] Rayner, N. A., Parker, D. E., Horton, E. B., Folland, C. K., Alexander, L. V., Rowell, D. P., Kent, E. C., & Kaplan, A., Global analyses of sea surface temperature, sea ice, and night marine air temperature since the late nineteenth century. *J. Geophys. Res.*, 108, 4407, (2003)

[23] Bellenger, H., Guilyardi, E., Leloup, J., Lengaigne, M., & Vialard, J., ENSO representation in climate models: from CMIP3 to CMIP5. *Clim. Dyn.*, *42*(7-8), pp.1999-2018., (2014)

[24] Taschetto, A. S., Gupta, A. S., Jourdain, N. C., Santoso, A., Ummenhofer, C. C., & England, M. H., Cold tongue and warm pool ENSO events in CMIP5: mean state and future projections. *J. Clim.*, *27*(8), 2861-2885., (2014)

[25] Hodges, K., Feature tracking on a unit sphere, *Mon. Wea. Rev.*, 123, 3458–3465, (1995)

[26] Hodges, K., Spherical nonparametric estimators applied to the UGAMP model integration for AMIP, *Mon. Wea. Rev.*, 124, 2914–2932, (1996)

[27] Hodges, K., Adaptive constraints for feature tracking, *Mon. Wea. Rev.*, 127, 1362–1373, (1999)

[28] Hoskins, B. J., & Hodges, K. I., New perspectives on the Northern Hemisphere winter storm tracks, *J. Atmos. Sci.*, 59, 1041–1061, (2002)

[29] Roberts, M. J., *et al.*, Tropical cyclones in the UPSCALE ensemble of high-resolution global climate models. *J. Clim.*, 2015, 28:574–596., (2015)

[30] Landsea, C. W., & Franklin, J. L., Atlantic hurricane database uncertainty and presentation of a new database format, *Mon. Weather Rev.*, 141, 3576–3592., (2013)

[31] Williams, K., *et al.*, Assessment of GA4/GL4/GO4/GSI4 and progress in addressing key model biases, Hadley Centre Technical Note, Sept. [Available at https://www.metoffice.gov.uk/binaries/content/assets/mohippo/pdf/c/l/additionalpaper_ga4_assessment.pdf], (2012)

[32] Haywood, J. M., *et al.*, The impact of equilibrating hemispheric albedos on tropical performance in the HadGEM2-ES coupled climate model, *Geophys. Res. Lett.*, 43, 395–403, (2016)

[33] Smith, D. M., Eade, R., Dunstone, N. J., Fereday, D., Murphy, J. M., Pohlmann, H., & Scaife, A. A., Skilful multi-year predictions of Atlantic hurricane frequency, *Nat. Geosci.*, 3, 846–849, (2010)
